# Comment on: Premenstrual and menstrual changes reported after COVID-19 vaccination: The EVA project

**DOI:** 10.1177/17455057221129395

**Published:** 2022-10-10

**Authors:** Tiago A Marques

**Affiliations:** 1 Centre for Research into Ecological and Environmental Modelling, The Observatory, University of St Andrews, St Andrews, UK; 2 Centro de Estatística e Aplicações, Departamento de Biologia Animal, Faculdade de Ciências da Universidade de Lisboa, Lisboa, Portugal

The recent paper in Women’s Health by Baena-García et al.^1^ got picked up by the mainstream Portuguese media as evidence of problems associated with COVID-19 vaccines. We live in a world where papers often make the headlines for the worst reasons. Scientists must be extra careful in how they communicate their findings and how they appropriately tone down their conclusions. As a scientist, I worry about the potential negative public perception about vaccines. Vaccines are overall safe and sound, and have saved millions of lives worldwide from COVID-19, but also many other diseases. This note voices my serious concerns about this study and its conclusions.

The paper lacks clarity regarding the way the sample was selected. The key statements provided in Baena-García et al.^1^ regarding the sampling are as follows: (1) “A cross-sectional study was conducted through an online survey.”; (2) “Data were collected retrospectively from women who had received the full vaccination course at least three months earlier . . . The online survey was open from June to September 2021.”; (3) “Women were asked about perceived menstrual changes in relation to pre-vaccination periods through Google Surveys”; (4) “Before starting the survey, participants accessed an informative text about the study aims and the average response time for the entire questionnaire, which stated that participation was completely anonymous and voluntary.” Detail is lacking to fully understand self-selection bias. The study claims to be a cross-sectional study. One does not need to go further than Wikipedia:

In medical research, social science, and biology, a cross-sectional study (also known as a cross-sectional analysis, transverse study, prevalence study) is a type of observational study that analyzes data from a population, or a representative subset, at a specific point in time.

The key point here is “a population, or a representative subset.” The sample considered by Baena-García et al.^1^ is not a representative subset of the population of interest. The statement about “online survey” leaves a reader wondering how respondents were directed to the survey, that is, what potential selection bias was involved. Regarding “perceived menstrual changes,” it is fair to say that women who are already mildly or strongly against vaccines would be more likely to answer and to be subject to unconscious bias. Finally, the survey would reportedly take about 20 min, a low estimate given the 45 questions, which deters answers from most people without some interest on the topic and/or a desire for some specific results that might support their own existing prejudices against vaccines. In particular, it is never stated explicitly if women were contacted to participate on the study or if they would self-enroll. If self-enrolling, how was that self-enrolling process. Did they received an email, a phone call, they were told about the survey just after the vaccine? Whatever the process was, the sampling process was clearly a convenience one.

When faced with, as in Baena-García et al.,^1^ a 45 questions survey about a given topic, one is much more likely to be going through the trouble of answering it if the topic is of interest to them or if a (self-assessed) effect was observed for oneself. Either way, estimates from such a convenience sample will be biased, with bias magnitude unknown. Simplistic interpretations derived from such data are necessarily flawed. Self-selection bias is ignored until section “Limitations and strengths,” and the extent of the likely impacts unassessed. Convenience self-selected sample studies should be avoided unless they are able to discuss at length the potential problems of such self-selection procedures, which Baena-García et al.^1^ did not. In conclusion, justified by Baena-García et al.’s^1^ own words,

. . . results are based on self-reported data provided by volunteers, which can result in a bias error (i.e. women who perceived changes in their menstrual cycle might have been more prone to participate). Therefore, the study sample was of convenience (i.e. women who voluntarily wanted to complete the survey), which could have affected the representativeness of the sample.

This study simply does not provide evidence for the fact that there are premenstrual and menstrual changes after COVID-19 vaccination. Given the small effects found, and the likely direction of the bias, one might even argue the paper supports the lack of a biological significant effect. I am not saying there are no effects of vaccination on menstruation, I am just saying that this study brings us no closer to find them if they are. To detect them if they are real, we need a proper randomized trial. In that I agree with:^1^ “Future studies are warranted to clarify the current prevalence of these disorders and the physiological mechanisms behind these . . .” Convenience samples do not allow reliable inferences (see also, for example, Andrade^2^) and should be avoided except for exploratory studies.

## Declarations

**Ethics approval and consent to participate:** Not applicable.

**Consent for publication:** Not applicable.

**Availability of data and materials:** Not applicable.
